# Microfluidic Multielectrode Arrays for Spatially Localized Drug Delivery and Electrical Recordings of Primary Neuronal Cultures

**DOI:** 10.3389/fbioe.2020.00626

**Published:** 2020-06-16

**Authors:** Giulia Bruno, Nicolò Colistra, Giovanni Melle, Andrea Cerea, Aliaksandr Hubarevich, Lieselot Deleye, Francesco De Angelis, Michele Dipalo

**Affiliations:** ^1^DIBRIS, Università degli Studi di Genova, Genoa, Italy; ^2^Istituto Italiano di Tecnologia, Genoa, Italy

**Keywords:** microfluidics, multielectrode array, drug delivery, *in vitro* electrophysiology, neuron

## Abstract

Neuropathological models and neurological disease progression and treatments have always been of great interest in biomedical research because of their impact on society. The application of *in vitro* microfluidic devices to neuroscience-related disciplines provided several advancements in therapeutics or neuronal modeling thanks to the ability to control the cellular microenvironment at spatiotemporal level. Recently, the introduction of three-dimensional nanostructures has allowed high performance in both *in vitro* recording of electrogenic cells and drug delivery using minimally invasive devices. Independently, both delivery and recording have let to pioneering solutions in neurobiology. However, their combination on a single chip would provide further fundamental improvements in drug screening systems and would offer comprehensive insights into pathologies and diseases progression. Therefore, it is crucial to develop platforms able to monitor progressive changes in electrophysiological behavior in the electrogenic cellular network, induced by spatially localized injection of biochemical agents. In this work, we show the application of a microfluidic multielectrode array (MEA) platform to record spontaneous and chemically stimulated activity in primary neuronal networks. By means of spatially localized caffeine injection via microfluidic nanochannels, the device demonstrated its capability of combined localized drug delivery and cell signaling recording. The platform could detect activity of the neural network at multiple sites while delivering molecules into just a few selected cells, thereby examining the effect of biochemical agents on the desired portion of cell culture.

## Introduction

Neurological and neurodegenerative conditions, such as Alzheimer, Parkinson disease, and multiple sclerosis, affect a large percentage of the world’s population. These diseases involve the progressive loss of neural functions due to a variety of factors such as oxidative stress, protein aggregation, or misfolding in the central and peripheral nervous system (Barnham et al., 2004). Given the variability and complexity of causes and symptoms, the ability to treat, study, and understand these conditions is limited to the tools capable of probing the signaling complexity of neuronal networks.

Multielectrode array (MEA) devices are widely used for investigating electrogenic cell connectivity, physiology, and pathology in brain tissues and in 2D neuronal cultures. MEA technology represents a unique electrical interface for cultured cells, as they can be directly grown on top of the electrodes (Spira and Hai, 2013). The key advantage of this technique is the capability to record and stimulate large populations of excitable cells, without inflicting mechanical damage to the cells (Hammerle, 1998). Nevertheless, this technique lacks the ability to provide a more comprehensive modeling of neuronal networks, missing the capability of manipulating neural activity at chemical level with high spatial resolution.

On the other hand, microfluidic devices are necessary and useful *in vitro* systems for acquiring a comprehensive view of pathology and disease progression by means of well-controlled and localized delivery of bio-chemical agents (Park et al., 2006). Moreover, these systems have the potential to improve therapeutic drug approval rates by providing more physiological and patient-specific *in vitro* assays. Although the complete *in vivo* complexity cannot be captured yet, these systems could help in recapitulating and simulating it in *in vitro* models. This technology permits to control the amount and distribution of fluid directly on the device recreating microenvironments at tissue, cellular, and molecular levels (Gross et al., 2007; Lin and Levchenko, 2015). The physiologic and pharmacologic response of complex cellular systems can be investigated with precise control of the environment surroundings by controlling the reagent and factor distribution via the microfluidic channels. This specific aspect is relevant in studies involving the effect of the treatment on specific parts of cell culture. By optimizing the device design, it is even possible to create spatial concentration gradients of molecules in the same culture (Tsur et al., 2017; Coluccio et al., 2019).

This approach is useful in cancer therapeutics research (Saadi et al., 2006), growth factors studies on neuronal stem cells (Chung et al., 2005) or more generally, because of the complex interactions occurring among neural cells in *in vitro* studies of neuronal networks (Blasiak et al., 2017; Kamande et al., 2019). Polydimethyloxane (PDMS) is widely used for allowing compartmentalization of the culture on the device and strictly limiting the communication between those separate environments through microfluidic channels (Takayama and Kida, 2016). In this field, creating co-cultures or inducing co-pathological cell cultures, where the unaffected cell population is in contact with other cell populations in a disease state, is an important tactic in neurodegenerative studies and pharmaceutical tests (Kunze et al., 2011). In fact, studying the disease progression pattern from the unhealthy cell population to the healthy one could provide the ability to monitor time-variant changes in cell network morphology and electrophysiology during disease progression. In the vast majority of cases, the current methods for analyzing the electrophysiological responses in these systems are done using fluorescence. However, these techniques are cumbersome and the attached fluorescent compounds could interfere with binding sites of interactive molecules, thereby hampering the conformational changes of the molecules and increasing non-specificity. In other cases, the evaluation is carried out by *a posteriori* methods that does not allow for simultaneous evaluation of the treatment and the disease evolution.

Therefore, combining microfluidic technologies with a MEA device would provide an immediate, label-free, and non-invasive procedure for investigating electrogenic cells response in the studied conditions. In this regard, Gladkov et al. (2017) designed two subpopulations of primary hippocampal neuronal cultures directly onto a MEA device with asymmetric channels. Morphological and functional connectivity between the two subpopulations could be analyzed thanks to the electrodes underneath the cell culture. Thereby, the unidirectional spiking pattern propagation through the microfluidic channels between the two sectors could be observed. These strategies provided powerful tools for advancing applications in therapeutics, diagnostics, and drug discovery.

Nevertheless, integrated and multiplex *in vitro* devices for neuronal modeling and disease therapeutics require the capability to achieve higher spatial resolution and tools for localized access to a specific cell environment (Pinto and Howell, 2007).

Despite the importance of this tool in medicine and therapeutics (Stewart et al., 2016), the selective and precise delivery of pharmaceutical agents including proteins, peptides, nucleic acid, or drugs into living cells remains a challenge in biosensing platforms.

In this context, the addition of nanostructures might increase the capabilities and specificity of a high throughput, label-free, non-invasive drug delivery. Different groups already focused on developing nanostructures such as gold nanotubes, nanopillar (Xie et al., 2012), and nanostraws (Xie et al., 2013) intended for highly specific and localized delivery of pharmaceutical agents on chip. In contrast with carrier mediated delivery methods, these nanostructures allow to carry molecules straight into the cells, enabling for a spatial, temporal, and dose-controlled delivery. Following this line, the N. Melosh group provided several advancements in the field. They culture cells onto nanostructured membranes forming tight cell–nanostraw interfaces (Xie et al., 2013). Cargoes are delivered from the fluid below the membrane, through the nanostraws directly into the cells, by either electrophoresis or diffusion. Their method supports an optimal delivery and ensures a low diffusion of the injected molecules into the external medium. However, the device does not allow for a direct electrophysiological evaluation of the cell response to the induced stimuli.

In this regard, further efforts were made in order to develop a functional approach that combines high precision cell response monitoring with a simultaneous molecule delivery. In previous work from our group, exploiting the multifunctional capabilities of hollow 3D nanostructures, we developed a tool able to monitor the bioelectrical activity of electrogenic cells while locally treating them with exogenous molecules (Cerea et al., 2018). Here, the hollow nanoantennas were used as a hybrid structure for intra- and extra-cellular recordings and as a nanoscale tool for selective and intracellular delivery. The microfluidic MEA (MF-MEA) device provided high quality intra- and extracellular recordings in human derived cardiac cells and in immortalized cardiomyocytes from mouse (HL-1). As a proof of concept, the simultaneous intracellular delivery was also verified with the localized injection of molecules in the cardiomyocytes through the 3D hollow nanotubes. However, the injected molecules were basic fluorescent dyes that had no effect on the spontaneous electrical activity of the cells, preventing us from correlating the effects of delivered drugs on the electrophysiological response of the culture. Moreover, the cardiomyocytes required only up to 4–5 days in culture for acquiring spontaneous electrical activity. Therefore, the experiments did not give indications on the full biocompatibility of the MF-MEAs for sustaining long-term cultures for several weeks, as it would be required for neurons.

In this work, we show that MEAs with microfluidic channels and protruding 3D nanotubes can sustain cultures of both hippocampal and cortical primary neurons up to their maturation. The neurons could be cultured for more than 3 weeks *in vitro* and showed the functional maturation of a network with spiking and bursting activity, ensuring the reliability of the device with neuronal cells. The cultures were characterized by fluorescence microscopy and by electrophysiological recording of the network activity. Further, the device demonstrates the ability to deliver selectively a stimulant molecule, caffeine, which dynamically affects the electrical response of the neuronal networks. Specifically, the neurons were chemically stimulated by localized injection of caffeine through the 3D nanotubes, while simultaneously monitoring the electrophysiological response of the network. As a result, we demonstrated the ability to observe the specific changes in electrical signaling of the treated neurons, while maintaining the physiological conditions in the rest of the culture.

## Materials and Methods

### Fabrication and Packaging of Microfluidic MEA

The MEA devices with microfluidic channels were fabricated with a technique explained in previous work by Cerea et al. (2018). Briefly, the membrane and nanochannels were fabricated on a 525 μm thick silicon wafer with 500 nm Si_3_N_4_ on both sides. A 400 nm Cr mask was sputter coated on one side in order to create nanochannels and membranes. Subsequently, a thin layer of photoresist was spin coated on top of it. The shape was defined with conventional UV photolithography (SÜSS MicroTec Mask Aligner) and subsequent Cr etching of the exposed areas. After dissolving the remaining photoresist in acetone, the wafer and the mask was exposed to reactive ion etching (RIE, SENTECH) in order to selectively etch the 500 nm layer of silicon nitride. After chemically removing the Cr mask, the 525 μm of silicon were wet etched in a solution of KOH and deionized H_2_O (1:2). After this procedure, the two 2 mm^2^ membranes were defined and the sample was immersed in distilled water to remove residues.

The gold coated 3D nanoelectrodes were fabricated as described in Dipalo et al. (2015). Briefly, a 2 μm thick layer of S1813 (MICROPOSIT S1813) was spin coated on top of the Si_3_N_4_ membrane. After the exposure and the following development in MF-319 (MICROPOSIT MF-319 DEVELOPER), 24 ti/au electrodes and conductive tracks were fabricated through electron beam evaporation. The unnecessary resist was lifted-off in hot remover-PG (MicroChem) and the residuals were removed by O_2_ plasma ashing. The 24 electrodes (100–900 μm^2^) were defined opening apertures on a 1.2 μm spin coated SU-8 photoresist in correspondence of each pad. The gold coated 3D nanoelectrodes were manufactured on the planar electrode through FIB (FEI NanoLab 600 dual beam system) lithography (with the exposure dose of 27 nC⋅μm^–1^) from the backside of the membrane, following the technique described in the work of De Angelis et al. (2013). The nanopillars decorate each electrode located on the Si_3_N_4_ membranes. These structures have an external diameter of 600 nm, an internal diameter of 150 nm, and a height of 1.2 μm. Each electrode contains an array with a variable number of nanostructures depending on the electrode dimensions and with a pitch of 4 μm between each nanostructure. The two membranes on the platform include eight nanostructured electrodes each. The finished device is mounted on a PCB and covered with a 2 mm thick layer of PDMS. The area with the electrodes is not covered in PDMS, allowing the confinement of the cells to the sensible area of the device and thereby reducing the number of cells per device. A glass ring was placed on top of the device in order to form a container for the cell medium. On the bottom side of the device, a thick rectangular piece of PDMS was plasma bonded, acting as a connector for inlet and outlet tubing to the microfluidic channels.

### Culture of Cortical and Hippocampal Neurons

First, each side of the MF-MEAs was sterilized under UV for 20 min. Next, the devices were pre-conditioned 2 days before cell seeding by overnight incubation at 37°C, 5% CO_2_, and 95% humidity in Primary Neural Growth Medium (PNGM), supplemented with 2 mM L-glutamine, GA-1000, and 2% Neural Serum Factor-1, in order to saturate the porous matrix of the culture well. Additionally, the tubing and microchannels at the bottom of the device were manually filled with PNGM using a sterile syringe. The following day, the PNGM was removed from the culture well to coat it with a solution of 30 μg/mL poly-D-lysine (Sigma–Aldrich) and 2 μg/mL laminin (Sigma–Aldrich) in phosphate-buffered saline (PBS) in order to enhance primary neuronal cells adhesion and proliferation on the devices. After coating, the device was again incubated for 4 h at 37°C, 5% CO_2_, and 95% of humidity. After that, the substrates were washed extensively with sterile water four times and dried in sterile conditions overnight before cell seeding. Rat cortex and hippocampus neuronal cells (Lonza Walkersville, United States) were seeded on the PDL-laminin substrates at a density of 500 cells/mm^2^ and incubated at 37°C, 5% CO_2_, and 95% humidity. Upon adhesion, after 2–2.5 h, PGNM was partially removed, leaving a small volume to ensure the cells do not dry, and fresh medium was added. Cultures were maintained for more than 3 weeks, while every 4 days, one-third of the medium was changed for fresh PNGM.

### Neuronal Network Activity Analysis

Data analysis was performed offline by using a custom software package named SpyCode (Bologna et al., 2010), developed in MATLAB (The Mathworks, Natick, MA, United States). Spike detection was performed by means of a threshold-based precise timing spike detection (PTSD) algorithm (Maccione et al., 2009). The algorithm requires three parameters: a threshold set to seven times the standard deviation of the baseline noise, a peak lifetime period (set at 2 ms), and a refractory period (set at 1 ms). To analyze and quantify the electrophysiological activity of cortex and hippocampal neuronal networks in both basal and caffeine-affected conditions, first order statistics were extracted. In particular, we evaluated the mean firing rate (MFR), i.e., the number of spikes per second from each channel, and the percentage of random spikes, i.e., the fraction of spikes outside bursts. Furthermore, burst detection was performed according to the method described by Pasquale et al. (2010). A burst is a sequence of spikes having an inter-spike interval (ISI, i.e., time intervals between consecutive spikes) smaller than a predefined reference value (set at 100 ms in our experiments), and containing at least a minimum number of consecutive spikes (set at five spikes). The parameters extracted from this analysis were the mean bursting rate (MBR) and the mean burst duration (MBD), which are, respectively, the frequency and the duration of the bursts at the single channel level.

Statistical analysis was carried out to determine significant differences between each sample pairs by using MATLAB. All data were presented as mean ± standard error of the mean. Since the data do not follow a normal distribution (evaluated by the Kolmogorov–Smirnov normality test), statistical analysis was performed using a non-parametric Mann–Whitney *U*-test.

### Pumping System

To maintain a constant flow, a commercial pressure-driven microfluidic system (MFCS-EZ, FLUIGENT) was used. The system was connected tightly to reservoirs containing the molecules we needed to inject. The flow rate was 100 μL min^–1^ to ensure a mild constant flow.

### Immunofluorescence Staining and Analysis, SEM Imaging

To acquire confocal fluorescence images, the culture medium was removed and the cell culture was washed using pre-heated PBS at 37°C. After washing, the cells were fixed through a 15 min incubation in paraformaldehyde (4% in PBS-1X) at room temperature (RT). Following fixation, cells were gently washed 4 times with 1X PBS, before permeabilization with 0.1% Triton in PBS for 15 min. Next, a blocking solution made of 5% FBS, 1% BSA in PBS was added for 30–45 min, before incubation with primary antibodies for 2 h at RT. Alpha Microtubule-Associated Protein (MAP2) polyclonal IgG (1:1000, Invitrogen PA5-17646) and alpha Glial Fibrillary Acidic Protein (GFAP) IgG2b (1:1000, Invitrogen PA1-9565) were used as primary antibodies and diluted in blocking solution. After primary antibody binding, the cells were washed three times for 5 min in PBS. Immediately after, the cells were incubated for 1 h in the dark at RT with the corresponding secondary antibodies marked with Alexa Fluor 488 and Alexa Fluor 546 (Life Technologies, Italy). To finalize, the cells were washed four times in PBS and the incubation with the nuclear marker DAPI (Invitrogen P36931) was performed for 15 min in the dark at RT. Images from rat brain neurons were acquired and visualized with 20X and 60X objectives using the Leica TCS SP5 AOBS Tandem DM6000 upright confocal microscope (Leica Microsystems, Germany).

To perform fluorescence analyses, the images were converted to grayscale to enhance the signal-to-noise contrast, followed by the setting of a manual threshold. The same threshold was applied to all the pictures used for the same type of analysis. The “*analyze particles*” function from ImageJ software (version Fiji) was used in order to count nuclei in images stained with DAPI and to count neurons and glial cells in images stained with MAP2 and GFAP. The particles were identified according to their sizes and shapes. In particular, the average size of the neuronal soma was taken into account in order to avoid under or over estimation of the cell number. The “*measure”* tool was used to evaluate the covered surface of neurites in images stained with MAP2. The values of interest were extracted from nine visual fields randomly chosen in each culture.

To perform scanning electron microscopy (SEM) imaging, cells were fixed at 10 days *in vitro* (DIV) with glutaraldehyde 2% solution in deionized water for 40 min at RT. Subsequently, the cultures were sequentially dehydrated by changing the solution every 5 min to a solution with a higher percentage of ethanol in water (from 30 to 100%). The samples were left to dry overnight in Hexamethyldisilazane (HMDS, Sigma–Aldrich). The dry samples were then coated with a 10 nm thick gold layer and analyzed by SEM (FEI NanoLab 600 dual beam system).

### Electrophysiological Recordings and Electroporation

The electrophysiological signals were acquired with a custom-made MEA acquisition setup based on a RHA2032 amplifier from the company Intan Technologies (Los Angeles, CA, United States). The electronic board provides recordings from 24 channels multiplexed on an analog output. The sampling rate is 10 kHz for each channel.

For cell electroporation, a pulse train of 2 V peak-to-peak (100 μs pulse duration, 20 Hz repetition rate) was applied for 10 s at the 3D hollow nanoelectrodes (Caprettini et al., 2017). Given the nanoscale nature of our 3D nanoelectrodes and the low voltage applied, the electroporation protocol allows to induce an effect limited to the cell(s) in immediate proximity of the nanoelectrode (Xie et al., 2013). After electroporation, we waited 1 min before recording the activity for 5 min.

## Results

### Microfluidic Multielectrode Array Sensor

The used platform was described in detail in the recent work of Cerea et al. (2018) and it is focused on the idea of designing a flow-through MEA sensor for simultaneous recording and localized drug delivery. The approach enriches the well-established passive MEA concept, with the possibility of cell poration and localized and carrier-free drug delivery.

**FIGURE 1:**
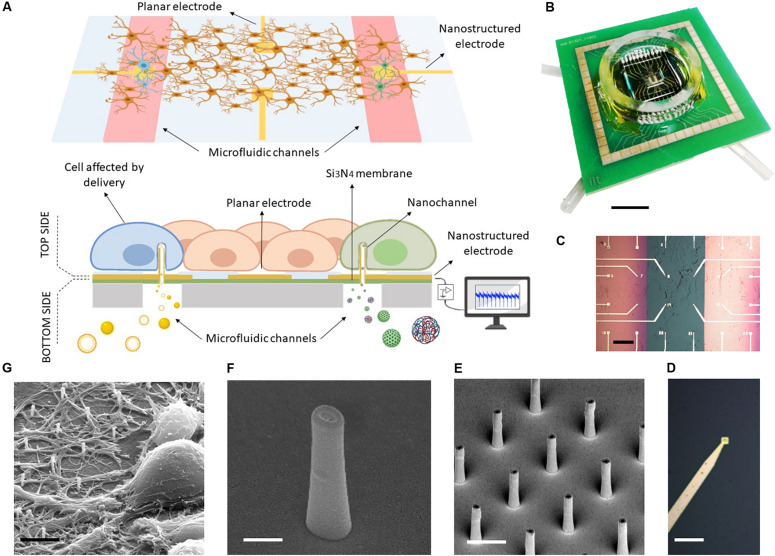
MF-MEA device. **(A)** Schematic of the device. **(B)** Top picture of the final device. Scale bar: 1 cm. **(C)** The top surface in detail, with feed lines and electrodes on thin silicon nitride membranes (pink) and bulk surface (green) Scale bar: 400 μm. **(D)** Magnification of an electrode. Scale bar: 30 μm. **(E)** SEM images of the hollow nanoantennas fabricated on the electrodes. Scale bar: 2 μm. **(F)** Magnification of a hollow nanostructure. Scale bar: 500 nm. **(G)** SEM image of neurons cultured on the nanostructures. Scale bar: 5 μm.

As depicted in Figures 1A,B, the MF-MEA is composed of a thin silicon nitride layer (green) on top of bulk silicon (gray) and two microfluidic channels that run underneath the surface. The 24 gold electrodes with an inter-electrode distance of 400 μm are fabricated and precisely distributed on the top surface of the device, comprising the two separated 500 nm thick silicon nitride membranes and the bulk section in between (Figure 1C). Each membrane is connected to a microfluidic channel and tubing which work as inlet and outlet for compound and molecule injection (see Figure 1B). In this way, we could store the compounds in these separate chambers (highlighted in red in the schematic Figure 1A) and deliver molecules simultaneously in separate parts of the cell culture.

The electrodes fabricated on the membranes (Figure 1D) are nanostructured with focused ion beam lithography (see Figures 1E,F). In particular, the MF-MEA configuration has eight of these nanostructured electrodes on each membrane and eight electrodes fabricated on the bulk section. The latter are considered as control. Several hollow nanoantennas protrude from each of these electrodes, providing an access from the lower compartment to the cells cultured Figon the top side (see Figure 1E). As described previously, these hybrid nanochannels allow for molecule delivery at their tip. The latter is in close contact with the cell membranes, thereby impeding the diffusion in other regions of the cellular network. The cellular medium will also not be contaminated by the fluid inserted into the microfluidic channels beneath, making it easy to wash the channels and switch to another molecule. A glass ring is attached on the device in order to contain the cellular medium when the cells are cultured on the surface (see Figure 1B). For completeness, Figure 1G shows an SEM image of a neuronal cell cultured and stained on such nanostructured electrode, following previously described protocols (Dipalo et al., 2017).

### Confocal Imaging of Neuronal Primary Cultures on Microfluidic Multielectrode Arrays

Hippocampal neurons were cultured at a density of 500 cells/mm^2^ on top of the MF-MEA platforms. These cells are considered standard models to investigate physiological properties of neurons, such as development, aging, and death, and represent a powerful tool to study degenerative disorders (Ghosh and Greenberg, 1995; Gärtner et al., 2006; Arnold et al., 2011; Gresa-Arribas et al., 2012). To investigate the cell viability and the development of the neuronal networks on the platform under physiological conditions, confocal fluorescent images of the neurons on the surface were obtained after fixation and staining at 18 DIV.

**FIGURE 2:**
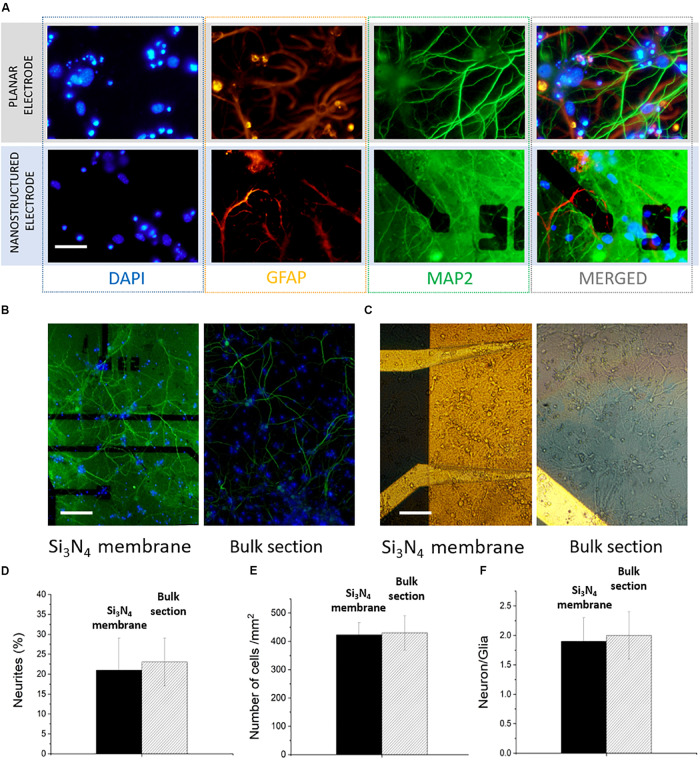
Confocal images of hippocampal neurons of microfluidic MEA. **(A)** Comparison between a planar electrode on bulk surface and a nanostructured one on thin silicon nitride membrane. Scale bar: 30 μm. **(B)** 20x image of the electrodes with microfluidic channel underneath (left) and the bulk surface (right). Scale bar: 100 μm. **(C)** 20x bright field images of the network on both surfaces. Scale bar 100 μm. **(D)** Percentage of neurites covering the device surface on the of bulk section and on the membrane. **(E)** Number of cells/mm^2^. **(F)** Comparison between the calculated neuron/glia ratios on the two sections of the device.

The major microtubule associated protein is visualized with MAP2 marker (green), while glial cells are visualized with a GFAP marker (orange). Additionally, the nuclei were stained with nuclear marker DAPI (blue). Monitoring the viability and the development of the neurons on both the bulk surface and the surface with the membranes was important for evaluating the possible effects of a heterogeneous surface and the presence of nanochannels. For example, the part of the neuronal culture situated on the microfluidic channels could be affected by different dynamics of growth factors due to the presence of a fluidic connection (provided by the hollow nanostructures) to the fluid beneath the thin membranes. In Figure 2A, highly magnified images of the cells on the electrodes are shown in order to highlight the viability of the neurons in the presence of both nanostructured electrodes on the silicon nitride membranes and planar electrodes on the bulk section.

The collective network development on both the silicon nitride membranes and the bulk material section was evaluated from 20X upright fluorescence and bright field images (Figures 2B,C and Supplementary Figure S1). The MAP2 expression shows a healthy and homogeneous neurite growth on both surfaces, with no marked distinctions. In particular, we quantified the area covered by neurites (MAP2) using the “*measure”* tool from the ImageJ software. The neurites covered 21 ± 8 and 23 ± 6% of the surface, respectively, on the microfluidic and bulk parts of the MF-MEA (Figure 2D). This indicates that the heterogeneous surface and the presence of nanochannels did not hinder the normal network development, suggesting that the platform design does not affect the cell culture adhesion, the network development, and the processes formation. We also estimated the number of cells per unit area using images of the nuclei stained with DAPI and the “*analyze particles*” function from ImageJ. As shown in Figure 2E, this resulted in a density of 430 ± 60 cells/mm^2^ in the bulk section and 423 ± 43 cells/mm^2^ on the microfluidic section of the device. We further calculated the neuron/glia ratio in the two cases using the same tools. The count was performed individually on GFAP and MAP2 stained images. The ratio was 1.9 ± 0.4 for the microfluidic region and 2.0 ± 0.4 for the bulk section, as reported in Figure 2F.

### Electrophysiological Recordings of Spontaneous Neuron Activity on MF-MEA

To support the applicability of the device to neuronal electrophysiology, we performed several experiments after culturing either primary hippocampal or cortical neurons from rat on our devices. As neuronal networks require approximately 3 weeks to reach maturation and bursting spontaneous activity, we started the recording sessions at 18 DIV (Ichikawa et al., 1993; Roppongi et al., 2017).

To examine the spontaneous electrophysiological activity of the network, the cells were kept at a controlled temperature of 37°C during recording. In order to preserve the sample sterility, a PDMS cap was placed on top of the glass ring. The so-constituted sensor was connected to the custom-made acquisition system that allows for acquiring signals from the 24 electrodes simultaneously.

**FIGURE 3:**
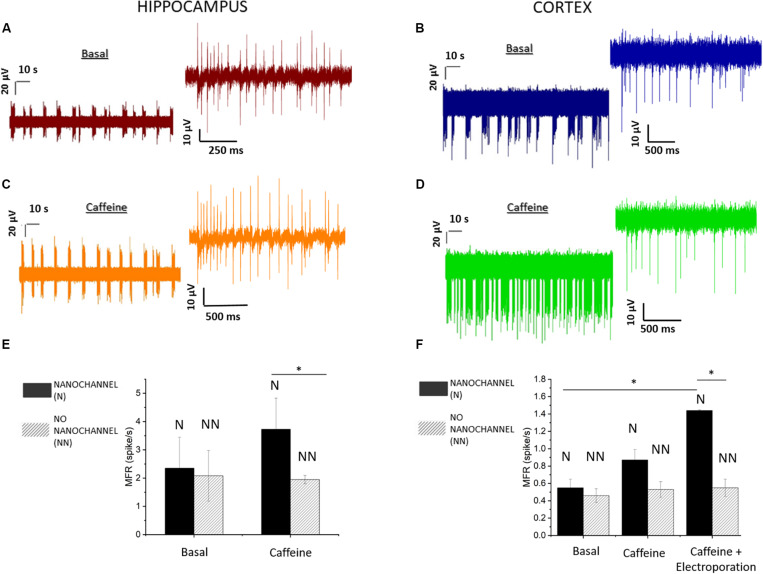
Hippocampal and cortical neuronal network recording and analysis. **(A)** Activity of hippocampal (20 DIV) neurons before and **(C)** after the caffeine injection. **(B)** Activity of cortical (21 DIV) neurons before and **(D)** after the caffeine injection. **(E)** Mean firing rate (MFR) of hippocampal neurons. Comparison between electrodes with (solid) and without (dashed) nanochannels in basal (*p* > 0.8) and caffeine conditions. **(F)** MFR in basal (*p* > 0.8), caffeine, and caffeine + electroporation conditions in cortical neurons for both electrodes (with and without nanochannels). Data are represented as mean ± SEM. Asterisks above the plots indicate statistical significance (*0.01 < *p* < 0.05).

The recorded activity showed temporal clusters and neuronal spiking patterns on the whole networks. We observed the behavior of the mature neuronal culture in physiological medium characterized by periodic firing and bursting activity. In particular, the analysis of MEA recordings indicates the presence of dominant spiking and bursting activity that occurred at the level of separated regions of the cultures, with no simultaneous activity taking place overall the complete cultures. This suggests that the neuronal networks presented separated clusters with little inter-cluster connectivity. Figures 3A,B depict 2-min long traces extracted from recordings on representative electrodes on the microfluidic channels, respectively, for hippocampal (20 DIV) and cortical (21 DIV) neuronal network activity. In total, we performed experiments on five MF-MEA devices. Three MF-MEA devices were used for cortical cultures, whereas the remaining two were used for hippocampal cultures. Each MF-MEA with cortical neurons was measured at 18, 21, and 22 DIV. Each MF-MEA with hippocampal neurons was measured at 19 and 20 DIV. The recordings lasted typically 5 min. The recorded extracellular spikes exhibited a good signal to noise ratio (SNR), allowing for a proper characterization of the bursting and firing activity of the cells on the electrodes. To analyze the electrical activity and the network’s dynamical properties from the obtained spike trains, the following metrics have been computed for both cortical and hippocampal neuronal networks: MFR, percentage of random spikes, MBR, and MBD. The above-mentioned parameters were calculated as means of the average values obtained from all the active electrodes that exhibited a minimum value of spiking and bursting rate of, respectively, 0.1 spikes/s and 0.4 bursts/min. The MFR is calculated through the sum between intra- and extra-burst spiking activities.

Cortical networks showed a MFR of 0.5 ± 0.1 spikes/s (mean ± standard error of the mean) and a MBR of about 3.1 ± 0.9 bursts/min. The hippocampal neurons exhibited a MFR and MBR of 2.2 ± 0.6 spikes/s and 16 ± 4 bursts/min, respectively. Regarding the burst duration, we found a MBD of 212 ± 23 ms for cortical neurons and 101 ± 16 ms for hippocampal neurons. We also evaluated the percentage of random spikes (i.e., the fraction of spikes outside bursts) for both culture types. In particular, cortical networks presented about 65.4 ± 2.1% of random spiking. The hippocampal neurons showed a level of random spiking of 15.1 ± 4.0%.

The calculated values are in line with those reported in literature for mature healthy rat cortical and hippocampal networks recorded *in vitro* (Cotterill et al., 2016; Napoli and Obeid, 2016). Therefore, as already suggested by our fluorescence analysis, the presence of microfluidic channels and pass-through 3D hollow nanotubes on the electrodes did not alter the correct maturation of hippocampal or cortical neurons. This implies the suitability of the MF-MEA device for culturing neuronal cells up to their maturation state and subsequent proper characterization of the network activity.

### Spatially Resolved Delivery of Caffeine

Next, we evaluated the capability of the MF-MEAs for inducing a selective chemical change in the electrophysiological behavior of sub-populations of neuronal cultures. The experiments consisted of delivering a stimulant molecule, caffeine, through the 3D hollow nanotubes fabricated on the microfluidic channels. Caffeine has multiple effects on the central nervous system, resulting in an increasing of neuronal activity. The main effects are mobilization of intracellular calcium, inhibition of phosphodiesterases, and antagonizing the adenosine receptors (Donato et al., 2003; Jackson and Thayer, 2006).

The hollow nanostructures enable a precise delivery in the few cells located on the nanostructured electrode area without affecting the remaining neuronal culture. Considering the typical cell dimension of 100–200 μm^2^ and the distance between the 3D nanostructures (4 μm), a single cell could typically interact with nine hollow nanostructures. Additionally, the branches from neighboring cells situated on this region could also be affected by the same delivery. Hence, a sub-population of neurons in immediate proximity of the electrodes are affected by the stimulation induced by the injected treatment. We have simulated the spatial and temporal dynamics of diffusion through the 3D hollow nanostructures. The results are reported in Supplementary Section S2. In particular, the simulations provide an estimate of approximately 40 μm as the maximum distance from the 3D nanostructure at which caffeine concentration is high enough to produce an effect. Therefore, we can consider the spatial resolution of the delivery to be within the area of the electrode itself. For comprehensiveness, in Supplementary Section S2, we also report further simulations of the molecule diffusion through the nanochannels in case of several starting concentrations (Supplementary Figure S2.4).

To assess the reproducibility of this selective delivery, the experiment was repeated on five different cultures. Caffeine, at a concentration of 16 mM in PBS, was first pre-heated at 37°C and thereafter inserted into the microfluidic channels below the MF-MEA. After filling the two separated compartments underneath the device, the caffeine molecules are able to diffuse across the nanotubes to the upper chamber where the cells are cultured. The caffeine was let to diffuse for 5 min in order to ensure a theoretical concentration of 2 mM delivered to the cells, in correspondence of the electrodes (Supplementary Section S2).

The effect was investigated by observing the firing/bursting rates of the affected cells before and after the administration of caffeine, and comparing them to the values related to the electrodes without hollow structures, where caffeine delivery did not occur. In Figures 3C,D, we report typical recordings after the caffeine injection on hippocampal and cortical neurons at 20 and 21 DIV, respectively. To evaluate the effects, we calculated and compared the MFRs before and after caffeine delivery, separating regions on the microfluidic nanochannels (affected by delivery) from the regions on the planar surface (no delivery). Figures 3E,F show the difference from the basal case in terms of firing rate between the electrodes with and without hollow nanostructures. Furthermore, for cortical neurons, bursting analysis was performed on the electrodes presenting bursting activity. We report the MBRs and MBDs before and after caffeine delivery in the treated regions in Supplementary Figure S3.

Both cultures show increased activity after caffeine delivery only in the parts of the culture laying on the microfluidic channels (Figures 3E,F). In particular, the MFR increases on the electrodes with nanochannels by 60% in case of hippocampal neurons and by 45% in case of cortical neurons. Differently, no statistical difference is observed on the electrodes without nanochannels. Hence, the caffeine delivery did not produce measurable effects on the neurons situated on the planar/bulk section of the MF-MEA, where no delivery occurred. The absence of increased activity in the central part of the cultures could be explained by the observations made in Section “Electrophysiological Recordings of Spontaneous Neuron Activity on MF-MEA,” related to the fact that the cultures presented a fragmented and clustered connectivity. In other cultures in which the neurons would be well connected over the complete networks, we expect that the increased excitability would be observed also in regions outside the microfluidic channels by propagation through the synaptic connections. As the device enables molecules delivery only in the area of the microfluidic channels without a significant diffusion in the cellular medium, it might be useful for high-localized pharmacological delivery studies. Lastly, we further report the calculated percentage of random spikes after caffeine delivery. Cortical networks present 43.9 ± 16.0% of random spiking, whereas the hippocampal neurons show a level of random spiking of 10.6 ± 3.1%.

In addition, we also investigated the effects of caffeine on the cortical neuronal network activity in case of cellular electroporation, assessing the possibility of an enhanced delivery of caffeine aided by membrane permeabilization. The electroporation protocol was applied to the neurons following the details in the methods Section “Electrophysiological Recordings and Electroporation.” In these experiments, we observed a further increase of the firing rate by approximately 50% after electroporation in comparison to the case of caffeine delivery without electroporation, as shown in Figure 3F. Thus, the effects of caffeine were enhanced by cell membrane permeabilization, without compromising the collective behavior, nor damaging the cell culture. In addition, in the nanostructured electrodes, the MBR rises from 4.8 ± 1.1 bursts/min (without electroporation) up to 8.1 ± 1.1 bursts/min with electroporation, with a percentage of random spikes of 36.3 ± 8.3%. The MBD changed from 281 ± 25 to 324 ± 26 ms with the addition of electroporation (Supplementary Figure S3). A possible explanation for these results might be related to the binding of caffeine to intracellular caffeine-sensitive gated channels (Meldolesi and Pozzan, 1998; Verkhratsky and Petersen, 1998). Sample recordings after caffeine delivery are reported in Supplementary Figure S4 with and without electroporation.

Similar experiments of membrane permeabilization by means of electroporation did not provide satisfactory results on hippocampal neurons, as no spontaneous activity could be recorded after electroporation. In this regard, further optimizations of the device design and of the electroporation protocol are needed to improve the success rate of membrane permeabilization on different cultures.

However, in perspective, the MF-MEA devices may also enable neuron membrane permeabilization by means of electroporation and simultaneous localized caffeine delivery to the network, without negatively affecting the neuronal cultures. This could be considered a first step toward internalization of non-permeant exogenous molecules or other specific materials into neurons using our platform. Given the possibility of simultaneous monitoring and localized injection, this approach might be useful in gene therapy, cellular reprogramming, or as an intracellular investigation probe.

## Discussion

In this work, we demonstrated localized drug delivery in and simultaneous electrical recording of primary cortical and hippocampal neurons using the MF-MEA platforms. Confocal fluorescence microscopy and electrophysiology experiments ensured the ability of the device to sustain cultures of primary neurons up to their full maturation without effecting their expected activity. The images confirmed a homogeneous network development overall the surface of the platform, without distinction between the thin nitride membranes decorated with 3D nanostructures and the planar surface. Furthermore, the maturity of the network was supported by the spontaneous bursting activity recorded from the electrodes of the MF-MEAs. The MF-MEAs also enabled the localized delivery of neuronal stimulants that chemically affected the electrical response of sub-populations in the network. The molecules inoculated from the nanochannels did not affect the parts of the cultures laying on the bulk MEA region, ensuring high spatial localization of compounds in the cell cultures. Therefore, the device allows for chemically treating only a selected sub-population of neurons, while simultaneously analyzing the whole network response.

Additionally, we showed the possibility of applying a non-invasive electroporation protocol to the cultured neurons with parallel pharmacological delivery, further paving the way toward effective studies on intracellular neuronal drug delivery on chip. In fact, the delivery of nucleic acids and other molecules to examine or manipulate cellular processes or gene expression on chip could improve preclinical research. Minimal effect on cell growth and differentiation is essential for advancing biological research, especially for neuronal cells. Subsequently, studying cells in their natural state, with spatial–temporal control, is extremely useful for examining and manipulating cellular processes and improve therapeutic drug potential.

At last, in this study, we focused on the devices’ ability for passive diffusion of molecules inside the nanochannels to achieve localized delivery to neurons. However, the MF-MEA configuration could also support the active delivery of charged molecules, using, for example, electrophoresis. In this regard, an external electric driving force for highly controlled spatial delivery of single molecules through 3D hollow nanoelectrodes was recently used with a thin membrane configuration resembling the MF-MEA (Huang et al., 2019). In this case, an external electric driving force applied between the upper and bottom microfluidic compartments promotes the diffusion of the molecule. This force is achieved by bringing an extra electrode in contact with the fluid in the bottom chamber. By applying a similar methodology to MF-MEAs, one might also enable active translocation of nanoparticles into single selected neurons using an applied electric field.

## Conclusion

We believe that the proposed platform could improve the studies of neuronal mechanisms under physiological and pathophysiological conditions. Specifically, in the first place, the delivered molecules are spatially separated from the cell culture, allowing simple insertion using a pumping system or syringes. Second, the method provides selective and high spatial resolution delivery of these compounds in the neuronal network under study. Third, the platform demonstrated promising preliminary results of soft neuronal electroporation, opening the way to studies on localized transfection of neuronal cells directly on-chip. Moreover, as future perspective, the device design presents flexibility for integration of additional features such as, for example, drug delivery enhanced by electrophoresis.

The above-mentioned key features make MF-MEA a promising device for studying dynamic cellular activities or alterations at multiple levels, enabling the observation of signal pathways during the selective inoculation of specific treatments. In fact, this will enable an easier and punctual manipulation of cell environment together with electrophysiological observations. For these reasons, the MF-MEA approach could be useful in basic neuroscience investigations, neurodegenerative diseases research, cancer therapeutics, or studies with neurons derived from human induced pluripotent stem cells (HiPSCs). Therefore, we believe that the MF-MEA will be useful for an improved understanding of the cellular mechanisms behind drug delivery, cell differentiation, disease progression, and treatment evolution at both network and single cell level.

## Data Availability Statement

The datasets generated for this study are available on request to the corresponding authors.

## Author Contributions

MD and FD conceived and planned the experiments. GB and AC carried out the samples preparation and experiments. NC and LD carried out the data analysis. GM performed the cell cultures and immunofluorescence protocols. AH performed the finite element simulations. All authors wrote the manuscript.

## Conflict of Interest

The authors declare that the research was conducted in the absence of any commercial or financial relationships that could be construed as a potential conflict of interest.
