# Supplementary material for: Microfluidic Multielectrode Arrays for Spatially Localized Drug Delivery and Electrical Recordings of Primary Neuronal Cultures
Source: Front Bioeng Biotechnol. 2020 Jun 16;8:626. doi: 10.3389/fbioe.2020.00626 (PMC7325920; doi:10.3389/fbioe.2020.00626)
Supplement: Supplementary file 1 [file Data_Sheet_1.docx]

**Supporting Information**

Microfluidic multielectrode arrays for spatially localized drug delivery and electrical recordings of primary neuronal cultures

Giulia Bruno, Nicolò Colistra, Giovanni Melle, Andrea Cerea, Aliaksandr Hubarevich, Lieselot Deleye, Francesco De Angelis* and Michele Dipalo*

S1.Fluorescence images

S2. Dynamics and evaluation of the amount of delivered molecules

S3. Bursting activity analysis

S4. Sample recordings of activity of cortical neurons

# **S1.Fluorescence images**


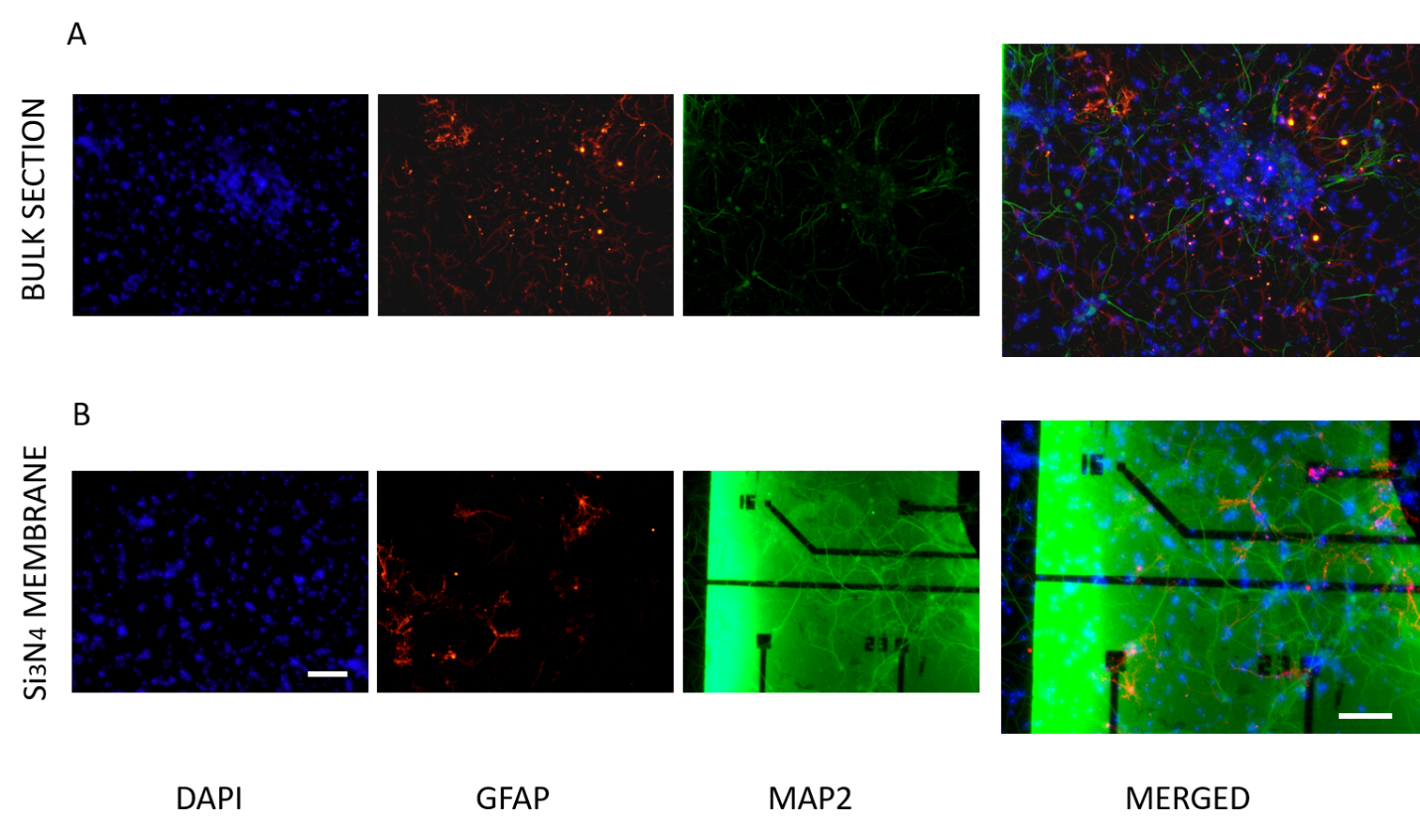


**Figure S1** A) 20x confocal fluorescence images of the bulk section of the device B) 20x confocal fluorescence images of the section with the thin silicon nitride membrane. Scale bar: 100 µm.

# **S2. Dynamics and evaluation of the amount of delivered molecules**

The caffeine solution flow has been modeled by means of the software COMSOL Multiphysics. A model from a previous work was used in order to simulate the spatial and temporal dynamics (Messina et al., 2015), modifying the parameters according to our system. A pure diffusion model has been considered to simulate the flow. The diffusion equation is given by

$$\frac{\partial c}{\partial t}+\nabla\cdot\left( -D\nabla c \right)=R$$

where c is the caffeine concentration, D the caffeine diffusion coefficient in Phosphate Buffered Saline (PBS), and R a source term. An estimate of the diffusion coefficient was derived by mean of Einstein-Smoluchowski relation:

$$D=\frac{k_{B}T}{6\pi\eta r}$$

Where k_B_ is the Boltzmann constant, T is the temperature, η is the PBS dynamic viscosity, and r is the caffeine average molecular radius. We considered the following values: T = 300K, η = 1×10^-3^ Pa·s, r = 1nm. The resulting value is D, 2.1×10^-10^ m^2^ /s. The equation is solved numerically by the Finite Elements Method (FEM) software COMSOL Multiphysics.

For the sake of clarity, the model calculate the diffusion of molecules without boundaries induced by the presence of cells or tensions induced by nanofluidics, which will decrease the mobility of the molecules and the velocity of the phenomenon. Thus, the estimated results could be considered as an upper limit of delivered molecules, concentration, speed and spatial resolution.

In order to understand the temporal and spatial dynamics of the molecules, we considered two different cases.

In the case of tight engulfment between the cell and the nanochannel, the molecules travel along the nanotubes and reach the cellular membrane in correspondence of the upper aperture, where they are blocked by the cellular membrane. A 1.2 µm tall antenna is considered, with diameter of the inner hollow region of 150 nm. Specifically, the molecules fill the nanotubes in 15 ms reaching a concentration of 16 mM , which interacts with the cellular membrane portion on top of the nanochannel (Figure S2.1). The calculated amount of delivered molecules in this time window is 340 molecules per nanotube.

***
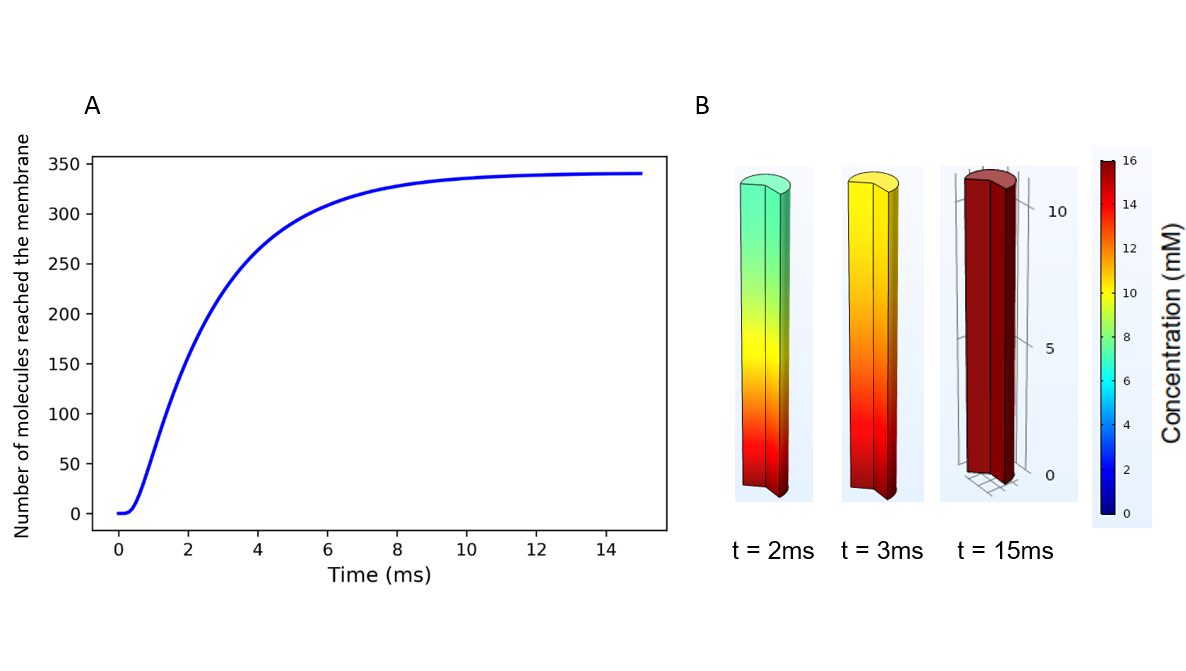
***

**Figure S2.1** Number of molecules that reaches the membrane as a function of time. B) Concentration of molecules inside the nanotube at 2, 3 and 15 ms.

In the other case in which not all the nanochannels are sealed with adhering cells, the molecules can flow into the upper cellular medium through the unoccupied nanotubes. Here, we can estimate the maximum distances of diffusion in specific time-windows. The area in which molecules diffuse out from the nanotubes is modeled as a half sphere with volume equal to 4/3·π·R^3^, where R is the inter-electrode distance of the MF-MEAs. After 300 s of diffusion, we could observe the dynamics and evaluate the concentration as a function of the distance (Figure S2.2A-B). From Figure S2.2A, we can observe that the concentration decreases to the µM range within 40 µm from the nanotube. For comparison, according to literature, a significant response from the cell can be observed with caffeine concentration in the order of hundreds of µM (Willson, 2018). Therefore, the diffusion from the nanotubes could be considered effective within the area of the electrodes.


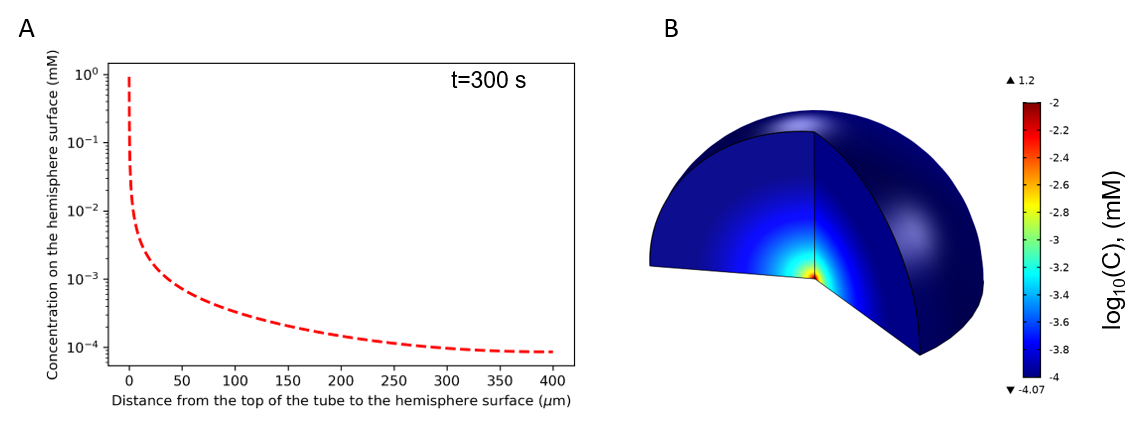


t = 300s

**Figure S2.2** A) Concentration of molecules with respect to the distance from the nanotube. B) False-color map of the average concentration in the considered volume.

In a further simulation, we calculate the concentration of the molecules on a hemisphere with radius 15 µm with respect to time. After 300 s, the average concentration reaches the value of 2mM, which is comparable with the one used in literature for stimulating cortical and hippocampal neurons (Willson, 2018) (Figure S2.3). This value corresponds to $8\times{10}^{9}$ molecules delivered from a single nanotube. Considering a linear trend of the concentration as function of time, the estimated diffusion velocity is $0.007 mM /s$ .

**Figure S2.3.** Simulated concentration of molecules as a function of time. After 300s, an average concentration of 2mM is estimated.

We also tested different starting concentrations of molecules in the reservoir to evaluate different delivery scenarios. In particular, we simulated the number of delivered molecules in case of initial concentrations of 2, 8, 16 and 24 mM up to 900 s of diffusion time. In Figure S2.4A, the number of molecules delivered as a function of time is reported at a distance of 400 µm distance from the nanotube. Figure S2.4B shows the distribution of molecules in space starting from the nanotube.


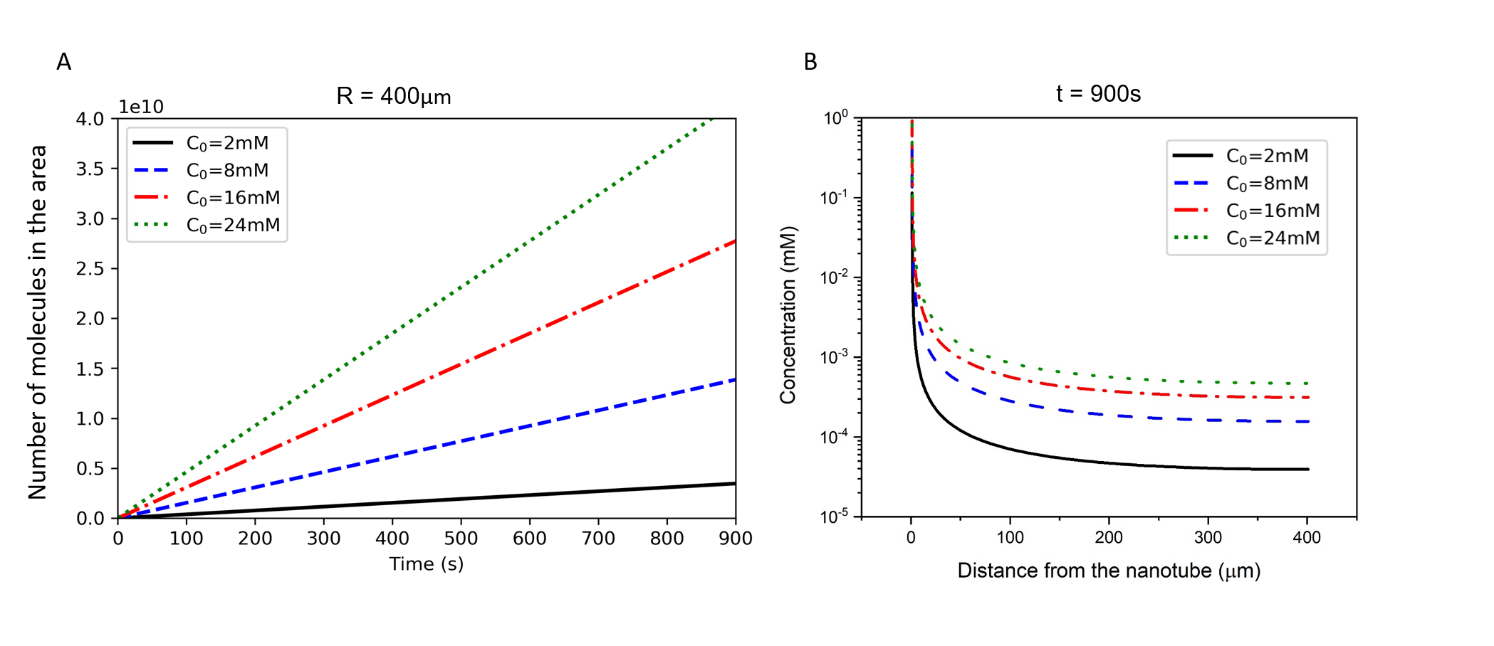


**Figure S2.4.** A) Simulated number of molecules for 4 different starting concentrations (C_0_) as a function of time in the area surrounding the nanotube with radius of 400 µm. B) Distribution of molecule concentration with respect to the distance from the nanotube after 900s of diffusion for the same starting concentrations.

# **S3. Bursting activity analysis**


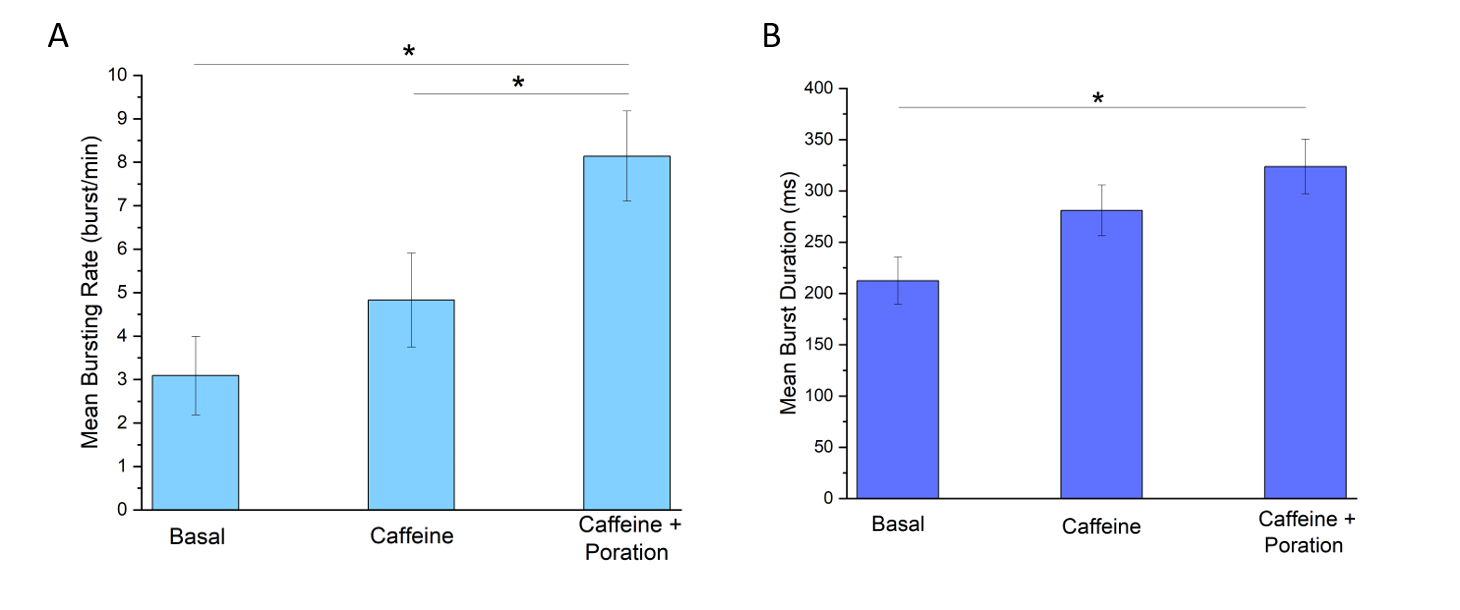


**Figure S3.** Burst activity of cortical neurons at 21 DIV in “basal”, caffeine” and “poration + caffeine” conditions. A) Mean Bursting Rate. B) Mean Burst duration. Data are represented as mean ± SEM.

# **
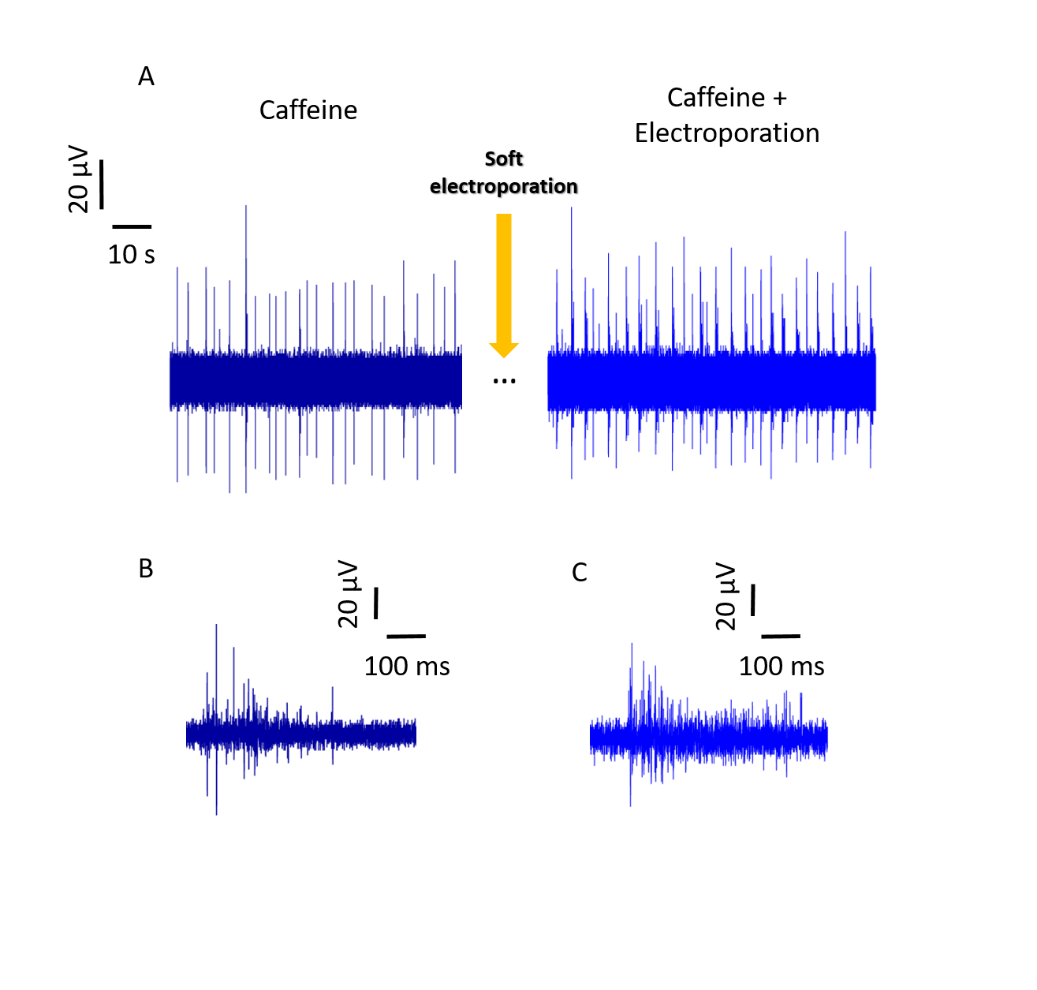
S4. Sample recordings of activity of cortical neurons**

**Figure S4.** A) Sample recordings of cortical neurons on MF-MEA after caffeine delivery and after electroporation. B-C) Magnification of the spiking and bursting events in caffeine case (B) and after electroporation (C).

**References**

Messina, G. C., Dipalo, M., Rocca, R. La, Zilio, P., Caprettini, V., Zaccaria, R. P., Toma, A., Tantussi, F., Berdondini, L., & Angelis, F. De. (2015). *Spatially , Temporally , and Quantitatively Controlled Delivery of Broad Range of Molecules into Selected Cells through Plasmonic Nanotubes*. 7145–7149. https://doi.org/10.1002/adma.201503252

Willson, C. (2018). The clinical toxicology of caffeine: A review and case study. In *Toxicology Reports* (Vol. 5, pp. 1140–1152). https://doi.org/10.1016/j.toxrep.2018.11.002
